# Munchausen syndrome mimicking refractory subcutaneous abscess with bacteremia, diagnosed by repetitive element sequence-based polymerase chain reaction: a case report

**DOI:** 10.1186/s13256-019-2212-7

**Published:** 2019-09-12

**Authors:** Naoki Iwanaga, Kazuko Yamamoto, Takahiro Takazono, Tomomi Saijo, Yoshifumi Imamura, Taiga Miyazaki, Koichi Izumikawa, Yoshihiro Yamamoto, Katsunori Yanagihara, Akira Yasuoka, Hiroshi Mukae

**Affiliations:** 1 Department of Respiratory Medicine, Nagasaki University Hospital, 1-7-1 Sakamoto, Nagasaki, 852-8501 Japan; 2 Department of Infection Control and Education Center, Nagasaki University Hospital, Nagasaki, Japan; 3 Department of Laboratory Medicine, Nagasaki University Hospital, Nagasaki, Japan; 4 Division of Respiratory Medicine, National Hospital Organization, Nagasaki Medical Center, Omura, Japan; 5 Department of Clinical Infectious Diseases, Toyama University Hospital, Toyama, Japan; 6 Division of Internal Medicine, Omura Municipal Hospital, Omura, Japan

**Keywords:** Munchausen syndrome, Recurrent cellulitis, Refractory infection, Fictitious injury

## Abstract

**Background:**

Rapid diagnosis and appropriate treatment of Munchausen syndrome is important not only for the patient but also for health care workers because a delay in diagnosis can worsen patients’ clinical outcomes, and result in a substantial medical cost.

**Case presentation:**

A young and previously healthy 24-year-old Japanese woman, a nurse, presented with complaints of refractory abscess on her left upper limb for 3 months. A physical examination on admission revealed low-grade fever and a subcutaneous abscess in her left forearm. Laboratory data suggested mild systemic inflammation and liver dysfunction, but no abnormalities of the immune system, including changes in the number of lymphocytes and neutrophils, neutrophil phagocytic capacity, and natural killer (NK) cell activity, were observed. A human immunodeficiency virus test was also negative. Multiple modalities, including positron emission tomography-computed tomography, failed to detect any cause and focus of infection except her left upper limb. *Streptococcus mitis* and *Prevotella buccae* were detected from the wound, but no microorganisms were detected in a blood culture. The cellulitis promptly resolved; however, exacerbation of the subcutaneous abscess with polymicrobial bacteremia repeatedly occurred unexpectedly. Because of this puzzling clinical course, the possibility of self-injury was finally suspected. Three syringes with needles, with a turbid liquid, were found in our patient’s bag. *Enterobacter cloacae* and *Enterococcus faecalis* were detected in the liquid, and an analysis via repetitive element sequence-based polymerase chain reaction determined that *Enterococcus faecalis* in the wound and syringe contents were genetically identical. She was diagnosed as having Munchausen syndrome and treated with the collaboration of a psychiatrist. She finally confessed that she had injected her own saliva and toilet water into the drip line and wound.

**Conclusions:**

This case report is valuable in that it is the first case in which this syndrome was diagnosed by a genetic method. Munchausen syndrome should not be neglected as a possible cause of refractory and recurrent infection.

## Introduction

Munchausen syndrome was named after Baron von Munchausen, who reportedly amused audiences with made-up stories in the eighteenth century. In 1951 Asher coined the term for persons seeking care for fictitious illnesses [1]. A recent review reported that the approximate prevalence of factitious disorder among psychiatric diseases ranges from 0.5 to 8% [2]. Typically, these patients have dramatic clinical findings and long histories of diagnosis and treatment at other institutions [3]. They are willing to receive diagnostic and therapeutic procedures, even if these are painful, and show great pleasure when receiving attention from medical staff [4]. The diagnosis of factitious disorder in *Diagnostic and Statistical Manual of Mental Disorders*, Fifth Edition (DSM-5) requires the following: falsification of physical or psychological signs associated with identified deception, presentation of themselves to others as ill, evident deceptive behavior, and behavior unexplained by another mental disorder [5]. However, it is generally not easy to raise this syndrome in differential diagnosis and to confirm a definite diagnosis.

Here we report an educational case of a young healthy woman with repeated subcutaneous abscess and bacteremia, subsequently diagnosed as having Munchausen syndrome. This case report is valuable in that it is the first case in which this syndrome was diagnosed by a genetic method.

## Case presentation

A 24-year-old Japanese woman presented to our hospital with complaints of recurrent fever and subcutaneous abscess on her left upper limb. She had been previously healthy and worked as a nurse in a general hospital. She had a history of multiple subcutaneous abscesses in a year, all occurring after surgeries of the shoulder and forearms performed for bone fracture and impingement syndrome. For 3 months before admission she had repeated episodes of subcutaneous abscess on her left forearm, which was the site of blood collection and arterial line puncture. Cefazolin, cefotiam, piperacillin, sulbactam/ampicillin, clindamycin, ceftazidime, gentamicin, and meropenem were sequentially administered, but the problem was not resolved. The cellulitis worsened in spite of these antibiotic treatments, and finally she developed bacteremia. Incision and drainage of her left upper limb was conducted 1 week before admission, and she was referred and admitted to our hospital for further investigation and treatment.

**Fig. 1:**
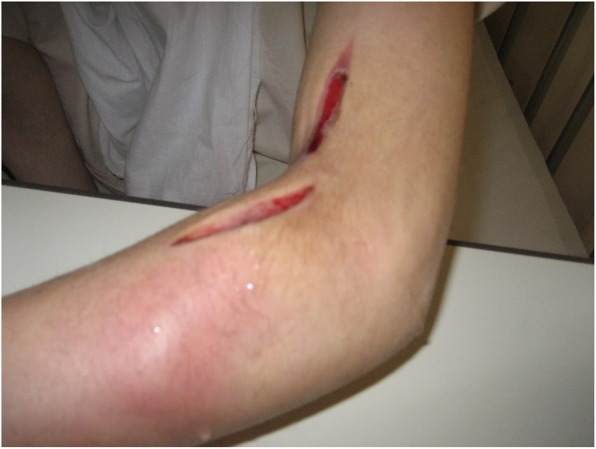
Appearance of the left upper limb on admission. The left upper limb was dissected for relaxation incision because of compartment syndrome due to progressive cellulitis. Redness, swelling, and induration of the skin by cellulitis were seen around the incision

She did not have underlying diseases or any family members with psychiatric disorders, autoimmune diseases, or malignancies. Her social history revealed no trouble with her surroundings, including her workplace. Her physical characteristics on admission were as follows: height, 155 cm; weight, 45 kg; body temperature, 37.4 °C; blood pressure, 122/76 mmHg; heart rate, 70 beats/minute and regular; and respiratory rate, 15/minute. A physical examination on admission did not reveal any focus of infection other than her left forearm. The skin of her left upper extremity had been incised at two points. Redness, swelling, and induration of the skin by cellulitis were seen around the incision (Fig. 1). Laboratory data suggested systemic inflammation and mild liver dysfunction (Table 1).

**Table 1:** Laboratory findings on admission

Hematology			Chemistry			Serology	
WBC	8400	/μL	CRP	2.63	mg/dL	HIV antibody	(−)
Stab	1.0	%	ESR	44.9	mm/h	STS	(−)
Seg	41.0	%	FBS	85	mg/dL	TPHA	(−)
Ly	38.0	%	HbA1c	5.3	%	HBs antigen	(−)
Aty-ly	1.0	%	IgG	1640	mg/dL	HCV antibody	(−)
Hb	10.8	g/dL	IgA	139	mg/dL	CMV-IgM	(−)
Ht	33.4	%	IgM	424	mg/dL	CMV-IgG	(−)
Plt	414	×10^4^/μL	IgE	7.4	IU/mL	CMV antigenemia	(−)
Chemistry			C3	152.0	mg/dL	EBV(VCA-IgM)	(−)
TP	8.1	g/dL	C4	28.8	mg/dL	EBV(VCA-IgG)	(−)
Alb	4.3	g/dL	CH50	50.9	IU/L	EBV(EBNA-IgG)	(−)
TB	0.3	mg/dL	Coagulation			ß-D glucan	(−)
AST	28	U/L	PT(INR)	1.15		Cryptococcus antigen	(−)
ALT	48	U/L	APTT	31.7	sec	Aspergillus antigen	(−)
ALP	339	U/L	Fib	568	mg/dL	Candida antigen	(−)
LDH	147	U/L	D-dimer	1.9	μg/mL	QFT-2G	(−)
CPK	19	U/L	Immunology			Microbiology	
BUN	6.0	mg/dL	NK cell activity		normal	Blood culture	(−)
Cr	0.59	mg/dL	NADPH oxidase		normal	Rapid influenza diagnostic test	(−)
Na	139	mEq/L	Reactive oxygen species		normal		
K	4.2	mEq/L					
Cl	102	mEq/L					
Ca	10.2	mEq/L					

*Alb* albumin**,** *ALP* alkaline phosphatase, *ALT* alanine aminotransferase, *APTT* activated partial thromboplastin time, *AST* aspartate aminotransferase, *Aty-ly* Atypical lymphocytes, *BUN* blood urea nitrogen, *Ca* calcium, *Cl* chlorine, *CMV* cytomegalovirus, *CPK* creatine phosphokinase, *Cr* creatinine, *CRP* C-reactive protein, *EBV* Epstein–Barr virus, *ESR* erythrocyte sedimentation rate, *FBS* fasting blood sugar, *Fib* fibrinogen, *Hb* hemoglobin, *HbA1c* glycated hemoglobin, *HBs* hepatitis B surface, *HCV* hepatitis C virus, *HIV* human immunodeficiency virus, *Ht* hematocrit, *INR* international normalized ratio, *K* potassium, *LDH* lactate dehydrogenase, *Ly* lymphocytes, *Na* sodium, *NADPH* nicotinamide adenine dinucleotide phosphate, *NK* natural killer, *Plt* platelets, *PT* prothrombin time, *QFT* QuantiFERON, *STS* serologic test for syphilis, *TB* total bilirubin, *TP* total protein, *TPHA Treponema pallidum* hemagglutination assay, *WBC* white blood cells

**Fig. 2:**
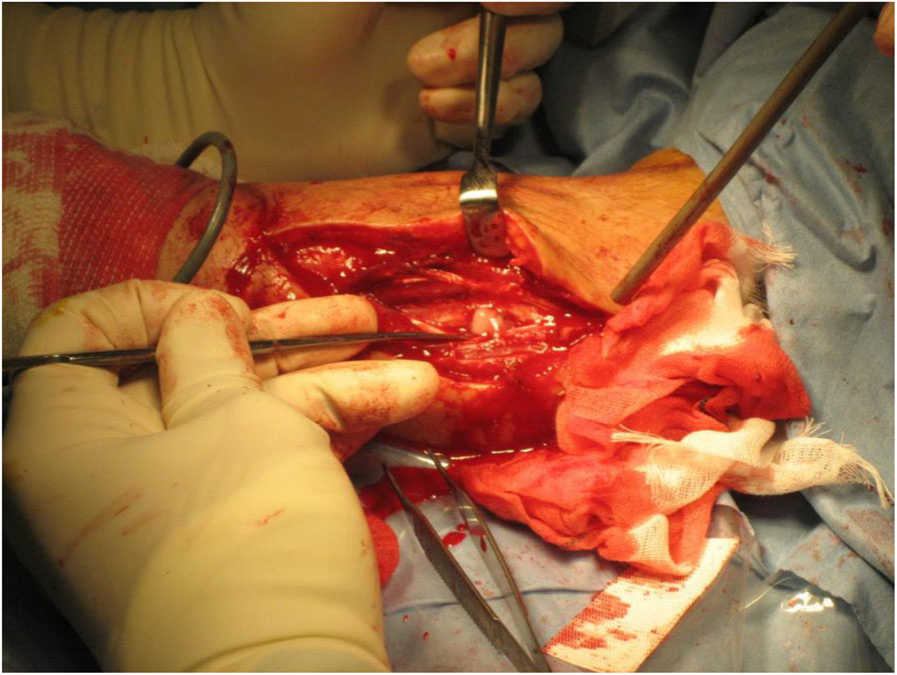
Emergency relaxation incision for alleviation of compartment syndrome in the patient’s left upper limb. Pus appeared in the supinator muscle

On admission, we considered the possibility of immunodeficiency, but there were no abnormalities of her immune system, such as changes in the number of lymphocytes and neutrophils, neutrophil phagocytosis disinfection capacity, or natural killer (NK) cell activity. She was also negative for human immunodeficiency virus (HIV) infection (Table 1). Contrast-enhancedcomputed tomography (CT) of her whole body, transesophageal echocardiography, magnetic resonance imaging of her heart and spine, and bone marrow puncture all failed to detect any focus of infection except her left upper limb. Microbiological examinations, including a culture for anaerobic bacteria using an anaerobic porter, detected *Streptococcus mitis*, *β-Streptococcus*, genus *Mobiluncus*, and *Prevotella buccae* from the wound, but no microorganisms were detected in her blood. At first, she was treated with meropenem (2 g/day) and linezolid (1200 mg/day) intravenously, and the cellulitis was promptly resolved. However, unexpectedly, she suddenly developed high fever and complained of severe pain at the site of the cellulitis. We made an emergency incision of her left upper limb for diagnosis of compartment syndrome and observed a jet of pus out of the supinator muscle (Fig. 2). As a result, the antibiotic was switched to garenoxacin (400 mg/day). *Stenotrophomonas maltophilia* was detected in blood and pus cultures, so sulfamethoxazole-trimethoprim was added. However, an allergic reaction to sulfamethoxazole-trimethoprim, such as acute fever and extensive skin eruption, occurred, so we switched to minocycline instead. Hyperbaric oxygen therapy was also performed nine times against the refractory soft tissue infection to improve neutrophil functions [6]. At the end, these multidisciplinary treatments allowed her to consider discharge, however, high fever and subcutaneous abscess of the same limb suddenly developed again.

**Fig. 3:**
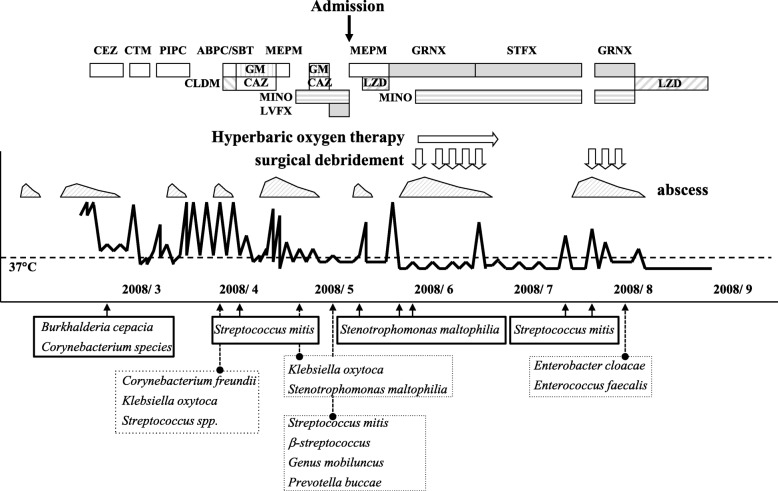
Clinical course of the patient. *Brackets with a solid line* show the culture of blood; those with a *broken line* show the culture of pus of wound. Intestinal bacteria and oral indigenous bacteria with polymicrobial pattern were detected from cultures of blood and pus samples. *Blank square*, β-lactam antibiotics. *Filled square*, quinolones. *Vertical line*, aminoglycosides. *Horizontal line*, tetracycline. *Shaded right line*, clindamycin. *Shaded left line*, linezolid. *ABPC/SBT* sulbactam/ampicillin, *CAZ* ceftazidime, *CEZ* cefazolin, *CLDM* clindamycin, *CTM* cefotiam, *GM* gentamicin, *GRNX* garenoxacin, *LVFX* levofloxacin, *LZD* linezolid, *MEPM* meropenem, *MINO* minocycline, *PIPC* piperacillin, *STFX* sitafloxacin

**Fig. 4:**
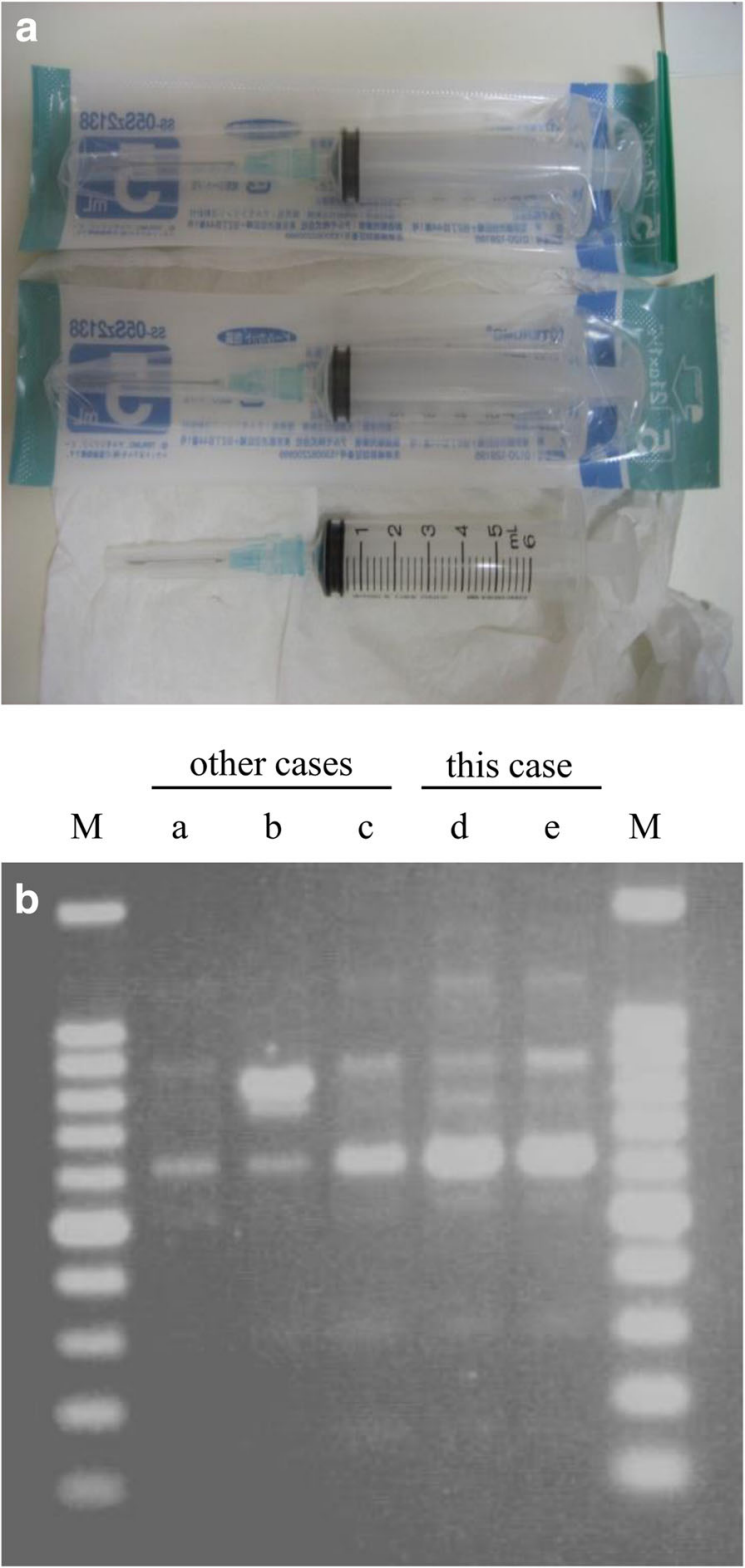
**a** Three syringes were found among the patient’s belongings, and one of these contained a small amount of turbid liquid. **b** DNA fingerprints generated from repetitive element sequence-based polymerase chain reaction analysis. The *Enterococcus faecalis* strains from pus derived from the muscle (*d*) and from the syringe contents (*e*) proved to be genetically identical, but strains from other patients (*a*, *b*, *c*) were different. *M* mark

As a result of this puzzling clinical course, especially the fact that multiple species, including oral indigenous bacteria with a polymicrobial pattern, were detected in cultures of blood and the wound abscess (Fig. 3), we finally suspected the possibility of self-injury. A psychiatrist was consulted and gave sufficient explanation to our patient and her family to persuade them to cooperate in diagnosing and treating her. Her sister found three syringes with needles in her bag, and one of these syringes contained a turbid liquid (Fig. 4a). *Enterobacter cloacae* and *Enterococcus faecalis* were detected in the liquid, with identical susceptibility to that of the bacteria detected in pus from the muscle. An analysis by repetitive element sequence-based polymerase chain reaction (PCR) determined that *Enterococcus faecalis* from the wound and the syringe contents were genetically identical (Fig. 4b). Finally, a diagnosis of Munchausen syndrome was confirmed.

No further episodes of fever and cellulitis occurred after the start of monitoring by camera and restraint of both upper and lower limbs but, 3 weeks later, our patient suddenly left our hospital and attempted suicide. An order was obtained for compulsory hospitalization for medical care and protection, and she was admitted to the psychiatric department. Two weeks later she confessed that she had injected her saliva or toilet water into a drip bag and into her wound with a syringe. She expressed feelings of extreme loneliness when deprived of care by medical staff. Her mental condition has gradually improved. She was discharged after 2 months of treatment in the psychiatric ward and was followed up in out-patients for a few years.

## Discussion

Munchausen syndrome should not be neglected as a possible cause of refractory infection with disproportionate clinical course. However, it is often difficult to raise the syndrome in differential diagnosis and, moreover, challenging to determine a definite diagnosis [7]. This is the first report showing that a PCR-based diagnosis could provide objective evidence for suspicious cases. Unnecessary diagnostic and therapeutic procedures can lead to irreversible morbidity and even death [8]. Experienced clinicians should consider the possibility of this syndrome earlier in similar circumstances; therefore, especially for primary physicians, this case report could have educational value.

Initially, we did not imagine that our patient would subject herself to the serious risks of self-induced infection and focused on treating her medical problems only from the physical aspect and failed to consider her mental health. We hypothesized that either the treatment had been insufficient or that she had a primary or secondary immune disorder as an underlying disease. There have been some reports of recurrent abscess formation in the same anatomic location [9]. However, she did not have any disorder affecting the function of her left arm, such as lymphedema due to axillary lymph node dissection or venous insufficiency, peripheral arterial disease, or co-infection with other pathogens such as dermatophytes [10]. We performed a variety of examinations to search for other infectious foci but could not find any. She showed no signs of underlying secondary immune disorders such as diabetes mellitus or HIV infection. Some reports have suggested that primary immunodeficiency may not be as rare in adulthood as previously thought, and that such disorders may occasionally escape detection until adulthood [11, 12]. Accordingly, we considered the possibility of neutrophil defects [13], but found no evidence to support this idea (Table 1). In the meantime, considering the resistance to various antibiotics, microbial examinations for fungus and multidrug-resistant bacteria were also conducted but failed to identify the cause.

As the results of multiple investigations were inconclusive, we began to suspect a psychiatric cause. We thought it might be significant that new complications and symptoms repeatedly appeared as soon as the test results were found to be good; this is one of the characteristics of factitious disorders [14]. We noted that she was usually in a good mood when her general condition was bad, but her mood worsened as her body recovered. Other factors included the fact that the focus of infection was confined to her left upper arm and she was right handed [4], and that the bacteria, normally resident in the oral cavity and intestinal tract, were atypical for wound infection [2], and that these microorganisms were diverse and produced polymicrobial sepsis [15]. In addition to these strange features, she was female, unmarried, and a healthcare worker [14]; all these features appeared in previous reports [16], and led to a suspicion of factitious disorder, especially Munchausen syndrome, due to her dramatic moods and the frequent switching of hospitals [17, 18]. However, a definite diagnosis of the disorder was extremely difficult. In the present case, the diagnosis was confirmed by the presence of three syringes with needles in our patient’s baggage, as similar cases reported before [19], and by the genetic conformity of the microbes from the syringe contents and the wound.

No definitive treatment for such patients has been established, but early therapeutic intervention by psychiatrists has been considered very important [20], and long-term psychotherapy has been reported to be effective to some degree [21, 22]. For those with underlying depression, anxiety, or psychotic disorders, medications can be helpful, although most patients deny the diagnosis and leave the hospital [23]. In our case, constant psychotherapy could be sufficiently helpful for her rehabilitation without any medications.

## Conclusion

We report a case of Munchausen syndrome presenting as refractory and recurrent infectious disease of subcutaneous abscess and sepsis. It needs to be emphasized that the diagnosis of this disease requires exclusion of hidden physical illness or psychogenic disorders. Rapid diagnosis and appropriate treatment could be important not only for the patient’s sake but also for health care workers, because delay in diagnosis can waste a great deal of money and time [24], and even endanger the life of the patient. In cases with fair suspicion of factitious disorders, a PCR-based diagnosis could be one source of objective evidence, easily allowing the patient to receive psychiatric treatment.

## Data Availability

All data generated or analyzed during this study are included in this published article.
